# Nanotheranostics Targeting the Tumor Microenvironment

**DOI:** 10.3389/fbioe.2019.00197

**Published:** 2019-08-14

**Authors:** Catarina Roma-Rodrigues, Inês Pombo, Luís Raposo, Pedro Pedrosa, Alexandra R. Fernandes, Pedro V. Baptista

**Affiliations:** UCIBIO, Departamento de Ciências da Vida, Faculdade de Ciências e Tecnologia, Universidade NOVA de Lisboa, Costa da Caparica, Portugal

**Keywords:** tumor microenvironment, nanomedicine, cancer therapy, diagnostic, gold nanoparticles, nanotheranostics

## Abstract

Cancer is considered the most aggressive malignancy to humans, and definitely the major cause of death worldwide. Despite the different and heterogenous presentation of the disease, there are pivotal cell elements involved in proliferation, differentiation, and immortalization, and ultimately the capability to evade treatment strategies. This is of utmost relevance when we are just beginning to grasp the complexity of the tumor environment and the molecular “evolution” within. The tumor micro-environment (TME) is thought to provide for differentiation niches for clonal development that results in tremendous cancer heterogeneity. To date, conventional cancer therapeutic strategies against cancer are failing to tackle the intricate interplay of actors within the TME. Nanomedicine has been proposing innovative strategies to tackle this TME and the cancer cells that simultaneously provide for biodistribution and/or assessment of action. These nanotheranostics systems are usually multi-functional nanosystems capable to carry and deliver active cargo to the site of interest and provide diagnostics capability, enabling early detection, and destruction of cancer cells in a more selective way. Some of the most promising multifunctional nanosystems are based on gold nanoparticles, whose physic-chemical properties have prompt for the development of multifunctional, responsive nanomedicines suitable for combinatory therapy and theranostics. Herein, we shall focus on the recent developments relying on the properties of gold nanoparticles as the basis for nanotheranostics systems against the heterogeneity within the TME.

## Introduction

As the knowledge about cancer development progresses, it highlights the complexity of the disease characterized by inter-tumor and intra-tumor heterogeneity between cancer types of different or the same anatomical region (Mroz and Rocco, 2016; Grzywa et al., 2017; Liu et al., 2018a). This interplay between growing tumor cells and surrounding environment creates a tumor micro-environment (TME), whose arrangement varies according to the anatomical region of the tumor and the genetic and phenotypic traits of cancer cells (Correia and Bissell, 2012; Abadjian et al., 2017). Still, several common features can be found, such as the micro-environment of epithelial tumors is generally composed by tumor cells, the extracellular matrix (ECM), stromal cells, including fibroblasts, mesenchymal stromal cells, cells from the blood and lymphatic systems, and occasionally adipocytes and cells from the immune system, including macrophages, T and B lymphocytes, and natural killer cells.

TME composition and maturation dictates tumor progression, prognosis and the efficacy of chemotherapeutics, where it is becoming more evident that one therapy does not fit all (Netea-Maier et al., 2018). That is why a thorough comprehension of a patient's TME may provide for important clues of the most effective therapeutics. For this, efforts have been made to develop imaging and therapeutic strategies focused on TME for improving the efficacy of treatment (Roma-Rodrigues et al., 2019). Perhaps the most innovative and effective strategies have been put forward by nanomedicine, that offers a vast number of diagnostics and therapeutics, alone or combined into a single platform, in what is known as nanotheranostics. The Medical Standing Committee of the European Science Foundation states that “Nanomedicine is the science and technology of diagnosing, treating, and preventing disease and traumatic injury, of relieving pain, and of preserving and improving human health, using molecular tools, and molecular knowledge of the human body.” The last decades have fueled the synthesis and assembly of a plethora of nanomaterials, such as nanoparticles made of noble metals, carbon, heavy metals, etc., in many forms, e.g., spherical or non-spherical nanoparticles, nanowires, nanotubes and nanofilms. These nanomaterials have got unique properties that might be explored for theranostics applications. For example, carbon nanotubes are excellent conductors, with exceptional strength; iron oxide nanoparticles are superparamagnetic; while gold nanoparticles have unique spectral (optical) properties (reviewed in Pedrosa et al., 2015; Elzoghby et al., 2016; Bhise et al., 2017; Bayda et al., 2018).

These nanostructures' primary advantages for biomedical applications are the small size within the same scale than that of biomolecules and an augmented area-to-volume ratio allowing an increased interphase area in a small mass (Pedrosa et al., 2015; Elzoghby et al., 2016; Bhise et al., 2017; Bayda et al., 2018). One crucial advantage of nanoparticles for cancer therapy is their tendency to naturally accumulate within tumors via enhanced permeability retention (EPR; Nichols and Bae, 2014). The fundamental features of EPR are hyperpermeability of tumor vasculature to large particles (enhanced permeability) and impaired lymphatic drainage, retaining the particles into the interstitial space of the tumor (enhanced retention) (Nichols and Bae, 2014). This way, nanomaterials passively accumulate at tumors' sites where they can then exert their therapeutic/diagnostic effect. In order to extravasate the vasculature and avoid renal filtration and liver capture, nanoparticles should range between 10 and 100 nm and preferably present a neutral or anionic charge (Danhier et al., 2010; Dreaden et al., 2012). Using the EPR to passively accumulate nanoconjugates at the tumor site has been thoroughly described in a general way. However, tumors are heterogeneous, impacting the capacity of nanoparticles to homogeneously penetrate the neoplastic tissue (Danhier, 2016). For example, larger particles (100 nm) have higher retentions times in the tumor (Danhier et al., 2010). Also, the EPR based accumulation varies greatly with the degree of vascularization, which is not always easy to predict (Danhier et al., 2010). There are also several tumor types that do not preset an EPR effect that can be used, such as prostate and pancreatic tumors (Danhier et al., 2010). Another limitation of passive targeting relying solely on EPR is the inability of passive targeting to access necrotic tissue, in the core of the tumor due to the low vascularization (Ngoune et al., 2016). These disadvantages can be overcome with different strategies, for instance, using drugs to modulate vascularization. For example, vasoconstriction drugs cause normal vessels constriction, but tumor vessels do not respond to this effect due to insufficient muscular structure, which leads to an increased uptake of particles by tumor tissues (Maeda, 2012). To overcome limitation imposed by using EPR alone, several strategies include active targeting by means of several moieties (e.g., antibodies or peptides), capable to promote ligand-receptor interactions at the surface of tumor cells, inducing receptor-mediated endocytosis and drug release inside the cell (Kobayashi et al., 2014). Such moieties include, for example, EGFR, TGF-α, folate, or glucose receptors, which are known to be overexpressed in cancer cells. Active targeting has been critical for the development of vectorization systems that enable the nanoparticles to deliver their cargo on tumor site improving the therapeutic effect (Dreaden et al., 2012). Among the variety of nanomaterials, spherical gold nanoparticles (AuNPs) have been extensively studied for cancer diagnosis and treatment, mostly due to their unique optical properties, easy synthesis in aqueous solution, and functionalization with biomolecules, which together have not presented toxicity to the cells and organisms (Conde et al., 2014). AuNPs can be easily functionalized with different moieties, such as drugs, targeting ligand, protein or peptides, nucleic acids, imaging agents, photosensitizers, bioactive/bio-responsive moieties, among others. Targeting ligands bound to the surface of nanoparticles interact with receptors selectively expressed in tumor cells (Haume et al., 2016). By means of targeting ligands, it is possible to profit from the EPR effect to accumulate the AuNPs in the tumor site and then direct the gold nanoconjugates selectively to cancer cells. This way the anti-tumor effect may be delivered solely (or mostly) to the malignant cells while sparing the healthy tissues, thus with a beneficial impact in decreasing side effects (Guo et al., 2017). In addition, bioactive/bio-responsive moieties can be sensitive to TME and respond to specific stimuli, such as pH or matrix metalloproteinase (MMP; Guo et al., 2017). Chemotherapeutics drugs may also be loaded onto or attached to the surface of nanoparticles, thus functioning as carriers (Singh et al., 2018).

Besides the possible functionalization of AuNPs to a specific TME target, naked AuNPs were found to disrupt the crosstalk between cells within the TME and, consequently, preventing tumor progression. For example, AuNPs were found to influence angiogenesis by diminishing both the tube formation and migration of endothelial cells, through blockage of vascular endothelial growth factor (VEGF) signaling from TME cells to endothelial cells (Zhang Y. et al., 2019). Also, AuNPs have been found to promote tumor vasculature normalization while increasing blood perfusion and reducing hypoxia (Li et al., 2017). These basic AuNP systems may also be used to improve imaging approaches to assist surgeons during tumor resection, by injecting functionalized AuNPs specifically into the tumor it is possible to distinguish between healthy and malignant cells (Singh et al., 2018).

## Tumor Microenvironment

**Figure 1:**
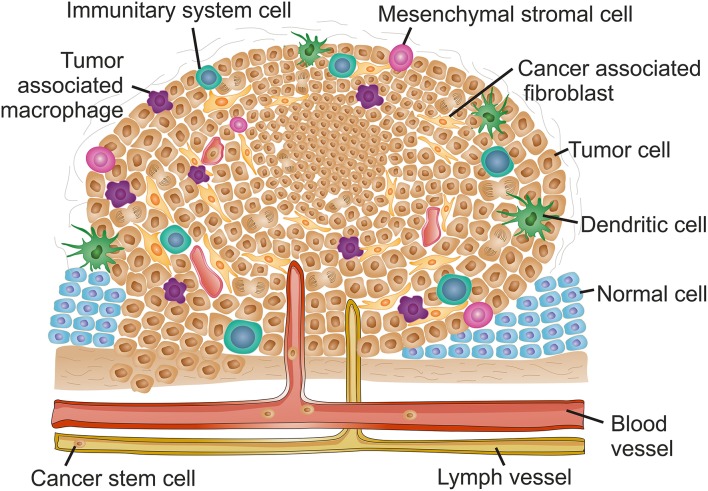
The complex context of tumor microenvironment. Schematic reorientation highlighting the diversity of elements within the tumor micro-environment (TME). The features and composition of a late stage solid TME are extremely heterogeneous with high intra- and inter-tumor variability. Common features include the appearance of hypoxic regions usually at the core of the tumor indicated as a denser cell population. Hypoxia is usually associated to decreased interstitial pH and induces angiogenesis, resulting in an uneven distribution of blood vessels along the TME. Lymphatic vessels are also frequently found in tumor late stages. The increased vasculature at the TME contribute for local invasion, the dissemination of cancer stem cells and formation of metastasis. The inflammatory environment recruits mesenchymal stromal cells and innate system tumor cells. Tumor associated macrophages usually infiltrate the tumor and promote a pro-inflammatory microenvironment contributing for tumor growth and reinforcing input for angiogenesis. Adaptive immune system cells are mainly found at the TME margins. The inflammatory environment stimulates transformation of fibroblasts into cancer associated fibroblasts that, together with an alteration of the extracellular matrix stiffness, contribute for increased desmoplasia at the TME and hence induce metastasis.

Despite the growing knowledge of tumor development and progression, it is virtually impossible to determine cause-effect chain of events, from the initial stage when cells become tumorigenic and initiate uncontrolled proliferation, to a mature high-grade tumor. Sometimes one is tempted to associate one event triggered by proliferating tumor cells to an event occurring within the TME, but this correlation falls far from reality since it is the evolving interplay of all TME components that ultimately will be responsible for tumor modulation and progression (Hanahan and Weinberg, 2011; Netea-Maier et al., 2018). Here we shall focus on each component of the TME separately for simplicity. However, it is crucial to understand that each one of these events, occurring for each component, may be triggered in a different way for a different tumor or tumor type, thus affect all the other TME components differently, and consequently resulting in different outcomes. Figure 1 and Table 1 highlight the major events occurring at the TME that contribute for tumor progression.

**Table 1:** Tumor microenvironment components and their major effects on tumor development.

**TME components**	**Major effect on tumor development**	**Disease prognosis**	**References**
Hypoxia	HIF activation	Poorer	Vaupel and Multhoff, 2018
	HIF loss of function	Poorer	Mazumdar et al., 2010; Lee et al., 2016; Nakazawa et al., 2016
	HIF mediated paracrine TME communication	Poorer	Huang et al., 2018; Sormendi and Wielockx, 2018
Aerobic glycolysis (Warburg effect)	TME acidification	Poorer	Lu, 2019
	Reactive Oxygen Species	Poorer	Gwangwa et al., 2018
	Genomic instability	Poorer	Gwangwa et al., 2018
	Activation of antioxidation defenses	Poorer	Gwangwa et al., 2018
Lymphoangiogenesis	VEGF secretion in TME	Poorer	Garnier et al., 2019
	Formation of lymph vessels by LECs	Poorer	Garnier et al., 2019
Activation of the Immune system	Impairment of anti-tumor immunity through LECs loss of function	Poorer	Farnsworth et al., 2019; Garnier et al., 2019
	M1-type monocytes activation through IFN-γ, etc.	Better	Italiani and Boraschi, 2014; Goswami et al., 2017; Jeong et al., 2019; Prenen and Mazzone, 2019
	M2-type monocytes (also known as TAM) activation through IL-4, IL-10, TGF-β, GM-CSF, Annexin A1, etc.	Poorer	Italiani and Boraschi, 2014; Goswami et al., 2017
Inflammation	TAM mediated chronic inflammation in TME	Poorer	Mantovani et al., 2018
	Activation of B and regulatory T lymphocytes	Better	DeNardo et al., 2010; Hui and Chen, 2015; Labiano et al., 2015; Wu, 2017; Steven and Seliger, 2018
	Activation of NK and NK T lymphocytes	Poorer	DeNardo et al., 2010; Hui and Chen, 2015; Labiano et al., 2015; Wu, 2017; Steven and Seliger, 2018
	GM-CSF and VEGF mediated production of MDSCs	Poorer	Schupp et al., 2017; Bruno et al., 2019
Altered ECM	Desmoplasia and metastatic dissemination	Poorer	Pickup et al., 2014; Kai et al., 2019
	Mesenchymal Stem Cells recruitment to TME	Either depending on TME	Trivanovic et al., 2016; Rivera-Cruz et al., 2017
	CAF differentiation through inflammation and TGF-β	Poorer	Liu T. et al., 2019; Sanford-Crane et al., 2019; Yoshida et al., 2019
Desmoplasia	Induction of EMT and the formation of Cancer Stem Cells	Poorer	Kang et al., 2019; Pearson, 2019; Vahidian et al., 2019
	Activation of MMPs in EMT	Poorer	Yao et al., 2018
Exosomes	Autocrine and paracrine communications between tumor cells and TME	Poorer	Hannafon and Ding, 2013; Franzen et al., 2014; Roma-Rodrigues et al., 2014, 2017a
	Autocrine and paracrine communications between normal cells and TME	Better	Hannafon and Ding, 2013; Franzen et al., 2014; Roma-Rodrigues et al., 2014, 2017a

*HIF, hypoxia induced factor; TME, tumor microenvironment; VEGF, vascular endothelial growth factor; LECS, lymphatic endothelial cells; IFN, interferon; TAM, tumor associated macrophages; IL, interleukin; TGF, tumor growth factor; GM-CSF, granulocyte-macrophage colony stimulating factor; NK, natural killer; MDSCs, myeloid-derived suppressive cells; CAF, cancer associated fibroblasts; EMT, epithelial to mesenchymal transition*.

The rapid proliferation of tumor cells results in a constrain in oxygen and nutrient supply that cannot be sustained by adjacent blood vessels (Hanahan and Weinberg, 2011). The oxygen scarcity experienced by growing tumor cells induce the cellular response to hypoxia, principally via hypoxia-induced factors (HIF) (reviewed in Vaupel and Multhoff, 2018). The HIF family of transcriptional factors is composed by HIF1, HIF2 and HIF3 proteins that activate genes involved in glucose metabolism, angiogenesis, cell proliferation and migration, and immune system modulation (Huang et al., 2017; Sormendi and Wielockx, 2018; Vaupel and Multhoff, 2018). The HIF response together with high energetic requirements, trigger a metabolic adjustment in tumor cells from oxidative phosphorylation to the aerobic glycolysis, in a process known as the Warburg effect (Gwangwa et al., 2018). This metabolic switch subsists even in the presence of oxygen and results in an increased secretion of lactate to the extracellular space and consequent TME acidification (Lu, 2019). The glycolytic metabolism and intensified proliferation of tumor cells result in an increase of reactive oxygen species (ROS) production, which in turn target cellular components, such as DNA, promoting genomic instability that further alters the cells' characteristics, and also induces the activation of antioxidant defenses (Gwangwa et al., 2018). These events, together with increased expression of efflux pumps for lactic and carbonic acid secretion gives an advantage to tumor cells to survive and thrive in hostile environments, contributing for multidrug resistance (MDR; Tsai et al., 2014). Interestingly, rather than oncogenic promoters in malignant cells, HIF proteins act as tumor suppressors (Mazumdar et al., 2010; Lee et al., 2016; Nakazawa et al., 2016). However, HIF-mediated paracrine communications between tumor cells and neighborhood, including stromal cells, immune system cells, metastases and extracellular matrix modulation, promote tumor development, rendering oncogenic features to HIF proteins at the TME level (reviewed in Huang et al., 2017; Sormendi and Wielockx, 2018).

The secretion of vascular endothelial growth factor A (VEGFA) by TME components, promote the sprouting of adjacent vessels through binding to VEGF receptors (VEGFR) located in endothelial cells (De Palma et al., 2017; Klein, 2018). The high levels of angiogenic signals at the TME, lead to the formation of vessels with defective or discontinuous basement membranes, resulting in the leaking vasculature with chaotic organization unevenly distributed along the tumor, with cancer regions enriched with vessels and cancer regions poorly supplied (Klein, 2018). This restricts the nutrient and oxygen supply to the TME, promoting hypoxia, and difficult the chemotherapeutic agents' distribution throughout the tumor (Klein, 2018). Moreover, the unbalanced distribution of blood vessels results in an altered distribution of cytokines involved in inflammatory and coagulation processes at the TME (Klein, 2018). Nevertheless, it is this less organized leaky vasculature that allows nanomedicines to passively target the tumor site.

The formation of lymphatic vessels at the TME, named tumor-associated lymphangiogenesis, is sustained by VEGF-C and VEGF-D secreted by tumor cells, immune cells, and other stromal cells (Garnier et al., 2019). Then, lymphatic endothelial cells (LECs) form single layer lymph capillaries with minimal basement membrane, that join to collecting lymphatic vessels with a basement membrane and valves that prevent retrograde flow (Garnier et al., 2019). The formation of lymphatic vessels at the TME is correlated with poor prognosis, since it favors metastatic propagation in distal organs (Garnier et al., 2019). On the other side, LECs have a prominent role in immune system modulation at the TME contributing for anti-tumor immunity (reviewed in Farnsworth et al., 2019; Garnier et al., 2019). Again, the defective lymph drainage assists the accumulation of nanomedicines at the locus via the EPR.

Concerning immune system cells, the TME varies considerably throughout the tumor development and throughout the various types of cancers (Netea-Maier et al., 2018). Due to the continuous changes and adaptations occurring at the TME, different recruitment factors (e.g., cytokines and chemokines) are secreted, resulting in the enrollment of cells from both innate and adaptive immune systems (Chen and Mellman, 2017; Clark, 2018). Importantly, the molecular signals composition of the TME determine the clinical outcome by promoting the tumor escape to immunosurveillance or tumor constrain (Netea-Maier et al., 2018). Once in the TME, monocytes can differentiate into two different types of macrophages depending on the chemical composition at the tumor location, the M1-type macrophages are formed in the presence of interferon gamma (IFN-γ), and the M2-type macrophages when exposed to different interleukins (IL, e.g., IL-4 or IL-10), transforming growth factor beta (TGF-β), Granulocyte-macrophage colony stimulating factor (GM-CSF), annexin A1 or tumor cell-surface molecules (reviewed in Italiani and Boraschi, 2014; Goswami et al., 2017). This macrophages polarization is crucial for tumor prognosis, as M1-type are correlated with a good prognosis, while tumor associated macrophages (TAMs) generally have the M2 phenotype and contribute for tumor growth, angiogenesis, invasion, and metastasis (Italiani and Boraschi, 2014; Goswami et al., 2017; Jeong et al., 2019; Prenen and Mazzone, 2019). Inflammation is usually displayed in TME, promoted initially by tumor cells (intrinsic pathway) and sustained and/or aggravated by other TME components (reviewed in Mantovani et al., 2019). A pro-inflammatory environment is usually accompanied by a poor prognosis (reviewed in Netea-Maier et al., 2018; Mantovani et al., 2019). The TAMs-mediated secretion of IL-1 cytokines contribute for chronic inflammation and strengthen a pro-tumoral micro-environment (reviewed in Mantovani et al., 2018). The role of the lymphoid lineage cells in tumor progression is also contradictory. While B cells and regulatory T cells create an immunosuppressive microenvironment, innate cytotoxic lymphocytes, natural killer (NK) cells and NKT cells contribute for an immunostimulant TME (DeNardo et al., 2010; Hui and Chen, 2015; Labiano et al., 2015; Wu, 2017; Steven and Seliger, 2018). In different types of cancers, the increased expression of GM-CSF and VEGF induce the production of myeloid-derived suppressive cells (MDSCs) at the bone marrow that are recruited to the TME where they remain undifferentiated (reviewed in Bruno et al., 2019). The presence of MDSCs is generally correlated with a poor prognosis, as they are involved in angiogenesis, and suppression of NK cells and CD8+ cytotoxic T cells (Schupp et al., 2017; Bruno et al., 2019).

In epithelial cancers, growing tumor cells and TME cell components are supported in an ECM with altered biochemical and biomechanical properties when compared with healthy tissue (reviewed in Pickup et al., 2014; Kai et al., 2019). The low oxygenation and inflammatory environment induce alterations in ECM proteins that result in desmoplasia, characterized by increased stiffness (Pickup et al., 2014; Kai et al., 2019). The ECM major contributors for desmoplasia include collagen types I, III, and IV, fibronectin, laminin, hyaluronic acid (HA) and osteonectin (Whatcott et al., 2011).

Stromal cells are also important for tumor development and prognosis. Mesenchymal stromal cells (MSCs) are recruited to the tumor due to the inflammatory environment and, according to the chemical composition at the TME they may promote or inhibit the tumor progression (reviewed in Trivanovic et al., 2016; Rivera-Cruz et al., 2017). Moreover, the inflammation and consequent secretion of TGF-β at the TME, induce the differentiation of fibroblasts into cancer associated fibroblasts (CAFs) (reviewed in Liu T. et al., 2019; Sanford-Crane et al., 2019; Yoshida et al., 2019). Besides tumor cells, CAFs are the most abundant cell type at the TME and play an important role in increased desmoplasia of the TME (Liu T. et al., 2019; Sanford-Crane et al., 2019; Yoshida et al., 2019).

Hypoxia, increased desmoplasia and the interactions between the several TME players favor the epithelial-to-mesenchymal transition (EMT) of tumor cells resulting in the formation of cancer stem cells (CSC) (reviewed in Kang et al., 2019; Pearson, 2019; Vahidian et al., 2019). EMT results in disruption of intracellular adhesion and loss of cell polarity, conferring migratory ability to CSC that could enter in adjacent blood or lymph vessels at the TME and travel to another anatomical location where they can experience mesenchymal-to-epithelial transition (MET) and potentiate the formation of a metastatic niche (Kang et al., 2019; Pearson, 2019; Vahidian et al., 2019). Matrix metalloproteinases (MMPs) have an important role in EMT, being responsible for the detachment of tumor cells from the ECM promoting CSC formation (Yao et al., 2018).

During the initial stages of tumorigenesis, the genomic profile of tumor cells determines the tumor maturation. As the cancer progresses, the intercellular communication between tumor and neighboring cells dictates TME and tumor progression, contributing for intra- and inter-tumor heterogeneity (Hanahan and Weinberg, 2011; Netea-Maier et al., 2018). Exosomes are pivotal for the cell to cell communication. Exosomes are endosomal pathway derived vesicles with 30–100 nm diameter, composed by a lipid bilayer with membrane proteins, entrapping soluble proteins, signaling molecules, including cytokines, chemokines and growth factors, and nucleic acids including mRNA and miRNA (Roma-Rodrigues et al., 2014). Importantly, exosomes' content depends on the cell of origin, often reflecting the physiological condition of the cell (Hannafon and Ding, 2013). After released to the extracellular milieu, exosomes can be internalized by secondary cells adjacent to the primary cell or travel through the vascular or lymphatic system to other anatomical location where they can be internalized by local cells. Once internalized, exosomes are able to modify the phenotype of the recipient cell that will adjust to the incoming signals (Franzen et al., 2014; Roma-Rodrigues et al., 2017a). Tumor cells derived exosomes (TCDEs) have an important role in tumor progression, including for example in immune system modulation, contributing in normal to tumoral transition of adjacent cells and preparation of the metastatic niche at a new anatomical location (Chen et al., 2017; Roma-Rodrigues et al., 2017a).

As discussed, TME development is astonishingly similar between types of cancer, exhibiting several common features in composition and organization. However, TME features are also highly dependent on tissue/organ. For example, hematological cancers will show an increased angiogenesis at the TME site in the bone marrow (reviewed in Cheng et al., 2018).

Notwithstanding the growing awareness of the role played by the TME in tumorigenesis, tumor progression and metastasis in the organism, most studies rely on *in vitro* data to build models, simulate conditions, and for the screening of novel drugs. What is more, most of these screenings and assessments are made using traditional cell lines that only resemble the real tumor in the individuals, letting the whole of intra- and inter-tumor heterogeneity at large. One of the most relevant trends in studying the effect novel diagnostics and therapeutic strategies for cancer has prompt for the development of realistic *in vitro* and *ex vivo* models to substitute the expensive and variable *in vivo* studies, thus allowing to speed up research in this critical field.

## Modeling the TME

Despite the amount of data from conventional *in vitro* models that rely on two-dimensional (2D) cell cultures, solid tumors are three-dimensional (3D) entities with cells growing on heterogenous gradients of nutrients and oxygen, suffering from various degrees of different chemical and physical stresses and interacting with different types of cells (LaBarbera et al., 2012; Weiswald et al., 2015). Therefore, promising drugs need to be tested in pre-clinical models mimicking the TME, and recurring to murine models with tumor xenografts, before they can get approval for human trials (Ranga et al., 2014). This is an expensive process that often fails to accurately predict therapeutic responses due to the fundamental differences between animal and human physiology. Only 5% of the compounds deemed promising in the initial evaluations are successful in clinical applications, which highlights the shortcomings of common standard models used for routine drug screening (LaBarbera et al., 2012; Ranga et al., 2014).

For assisting faster and more robust clinical translation, several (cell) models have been proposed. For example, humanized mice (HM) have been increasingly used to reduce the gap between animal and humans. HM are genetically engineered mice with human genes or human cells alongside the tumor xenografts (Morton et al., 2016). Another increasingly used pre-clinical model is patient derived xenografts (PDX) (Hidalgo et al., 2014). Mice xenografts produced directly from tumor biopsies can be used to more accurately predict how patients will respond to treatment and opens the door to personalized therapeutics (Hidalgo et al., 2014). However, these animal models are too expensive for high throughput screening (HTS) of novel drugs or treatment vectorizations (Rodrigues et al., 2018; Sasmita and Wong, 2018). For a long time, HTS has strongly depended on primary screening made with 2D cultures whose physiological and TME associated limitations have been surpassed by the advent of 3D cell culture models. For instance, cellular signal transduction pathways have been shown not to properly be activated in 2D cultures. Moreover, sensitivity to chemotherapeutic agents and induction of apoptosis are impaired in cell lines grown in 2D monolayers due to the lack of cell-cell and cell-extracellular matrix interactions, that are present in tissues and in 3D models (Kenny et al., 2007; Serebriiskii et al., 2008; Yin et al., 2016; Reynolds et al., 2017; Riedl et al., 2017; Buzzelli et al., 2018). Also, these models made possible the study of angiogenesis within a tumor and the evaluate the importance of the immune system in tumor growth (LaBarbera et al., 2012; Ranga et al., 2014; Buzzelli et al., 2018).

The simplest 3D model are multicellular tumor spheroids, or simply spheroids, obtained when adherent cell lines are grown in non-adherent conditions. The cells form 3D structures similar to *in vivo* tumors and are capable to recapitulate the TME interactions that are crucial for *in vivo* development (Sutherland et al., 1971; LaBarbera et al., 2012; Lancaster and Knoblich, 2014; Ranga et al., 2014). At first, spheroids were grown with only one cell line, however the complex nature of solid tumors led to the development of more intricate systems, with two or more different cell types, and thus able to mimic ECM, inter-cellular signaling and *in vivo* growth (LaBarbera et al., 2012). These more complex spheroids, with at least one tumor cell type, have been called organoids. Although the term was originally developed for spheroids grown from stem cells or organ progenitor cells, that would grow and differentiate into different cell types resulting in a spheroid with spatial auto-organization of different cell types similarly to the organization found *in vivo* for the organ from which they derive (Sung et al., 2008; LaBarbera et al., 2012; Lancaster and Knoblich, 2014). Considering that, in this filed, terminology is not consensual, we shall name spheroids to those multicellular spheroids constituted with only one cell type; multitype spheroids with at least one tumor cell line/type will be named tumor organoids, while the others will be named normal-type organoids.

In order to devise 3D systems that more accurately reflect the molecular diversity of tumors and their respective TME, there was a need for protocols to promote the growth of spheroids and/or tumor organoids derived from cancer biopsies from patients (Song et al., 2018; Vlachogiannis et al., 2018). These patient derived tumor organoids are capable to recapitulate the molecular profile of the patients' tumors and have become a valuable platform for HTS of compounds and for personalization of therapy (van de Wetering et al., 2015; Vlachogiannis et al., 2018). For example, AuNP have already been tested in some of the novel systems constituted by A549, HEG2, S2PV10, HCT116, and MCF7 cancer line spheroids so as to evaluate NP penetration within the tridimensional structure (Huang et al., 2012; England et al., 2013; Rane and Armani, 2016). Also, the potential photothermal therapy effectiveness of AuNPs have been evaluated on HeLa, normal ovarian cells, and human umbilical vein endothelial cells (HUVEC) spheroids, and in HeLa tumor organoids (Lee et al., 2018). Additionally, normal-type organoids have been used to test potential nephrotoxicity of AuNPs (Astashkina et al., 2014).

To further narrow the gap between *in vitro* and *in vivo* models, great efforts have been made to use microfluids platforms to reproduce more accurately TME and normal tissue functions, originating tissue-on-chip and organ-on-chip systems. The first organ-on-a-chip models (OoC) presented cells capable to replicate the physiological functions of the lung and heart within a microfluidic device with appropriate channels, chambers and mechanic movement (Huh et al., 2010; Annabi et al., 2013). The combination of different cell types, from liver and skin, within a multi-organ-chip, proved their potential in therapeutic drug testing (Wagner et al., 2013). The use of cancer cell lines on microfluidic devices led to the construction of tumor-on-a-chip models (ToC), also called cancer-on-a-chip. These systems have been shown to accurately mimic several features observed in tumors, such as pressure, chemical and gas gradients and fluidic shear stress, while being more amenable to HTS than other previous 2D and 3D models (Bhatia and Ingber, 2014; Tsai et al., 2017; Sleeboom et al., 2018; Shang et al., 2019). These ToC have been successfully used to co-culture cancer cells with endothelial cells, thus facilitating the study of the interplay in the TME between vasculature and tumor cells (Sleeboom et al., 2018; Shang et al., 2019). Also, these chip-based platforms have been used to assess nanoparticles' fate and action in more complex models. For example, AuNPs have been tested in an OoC with HUVEC to evaluate how endothelial thermotolerance could affect nanoparticle transport to tumors (Bagley et al., 2015). ToCs incorporating spheroids of MDA-MB-453 and MCF-7 cells were used to study tissue penetration and cellular uptake of AuNPs (Albanese et al., 2013; Kwak et al., 2014). Deeper insights into the EPR effect have also been possible via a tumor-vasculature-on-a-chip developed to study the perfusion of nanoparticles in an *in vitro* model (Wang H. F. et al., 2018).

It is based on these developing 3D complex models, harboring different players from within the TME, that several nanomedicines have been tested and evaluated, aiming at eventually being translated to the clinics and providing additional tools for the fight against cancer. Not surprisingly, these complex 3D and chip models have been crucial for the evaluation of innovative approaches in tacking the TME, which, whole or in part, make use of the one or more of the elements within the TME that modulate tumor growth and development.

## Targeting TME via Gold Nanoparticles

Several types of nanomaterials, and nanoparticles in particular, have been proposed as tools to study and/or assist in the fight against cancer. Amongst these nanoparticles, gold has emerged as a material of choice due to their spectral properties, ease of synthesis and functionalization. Additionally, small (15–60 nm) spherical AuNPs has not been shown to exhibit toxicity, which has prompt for their use as valuable platforms for diagnostics (e.g., imaging) and therapy. The interaction between AuNPs and the TME is, thus, of crucial relevance toward the development of innovative efficacious nanomedicines that could tackle tumors alone or in combinatory approaches with more traditional therapies. Table 2 summarizes the specific characteristics of the TME to be targeted by AuNPs for diagnostic and/or therapy in cancer.

**Table 2:** Targeting strategies for AuNPs toward TME.

**System name**	**Nanoparticle (type)**	**TME component**	**Diagnostics**	**Therapeutics**	**3D *in vitro*/*in vivo* model**	**References**
“Smart” AuNPs (SANs)	AuNP	Acidic pH	PAI		Xenograft mouse model	Song et al., 2016
c(RGDyK)-MHDA/LSC@AuNP	AuNP	Acidic pH	PAI		Xenograft mouse model	Li et al., 2019
LGAuNP	AuNP	Acidic pH	FLimaging		Xenograft mouse model	Lai et al., 2017
GNPs-CKL-FA	AuNP	Acidic pH	FLimaging		Xenograft bearing mice model	Tang et al., 2019
AuNR@ MSN-RLA/CS(DMA)-PEG	AuNR	Acidic pH		PDT; PTT	Xenograft bearing mice model	Liu et al., 2018b
DOX-EGF-SA-AuNP	AuNP	Acidic pH		DD	Xenograft mouse model	Feng et al., 2017
MC-GNPs	AuNP	Acidic pH		PTT		Li et al., 2014
pH-GSNPs	Gold Shell nanoparticles	Acidic pH		PTT; DD		Dai et al., 2015
MBA/SMART-AuNP	AuNP	Acidic pH	SERS imaging	PTT		Jung et al., 2013
Gold nanomachines	AuNP	Acidic pH	PAI	PTT	Xenograft mouse model	Yu et al., 2017
AuNC@MnO2	AuNC	Acidic pH; Hypoxia; Immunosuppressive tumor microenvironment	FL imaging; PAI; MRI	PDT	Xenograft mouse model	Liang et al., 2018
V7-CMG	AuNR	Acidic pH	MSOT	DD	Xenograft mouse model	Zeiderman et al., 2016
Nanoprobe	AuNP	MMP-2; MMP-7	FLimaging			Wang et al., 2012
MMP-sensitive AuNP probe	AuNP	MMP	NIRF tomographic imaging		Xenograft mouse model	Lee et al., 2008
MMP-GC-AuNPs	AuNP	MMP	CT; FLimaging		Xenograft bearing mice model	Sun et al., 2011
G-AuNPs-DOX-PEG	AuNP	MMP-2 Acidic pH		DD	Xenograft bearing mice model	Ruan et al., 2015a
DOX-GLT/EGCG AuNPs	AuNP	MMP	FLimaging	DD		Tsai et al., 2016
G-AuNPs-DC-RRGD	AuNP	MMP-2; Acidic pH	FLimaging	DD	Xenograft bearing mice model; Spheroid	Ruan et al., 2015b
DOX-substrate/AuNP	AuNP	MMP-2	FLimaging	DD	Xenograft bearing mice model	Chen et al., 2013
CDGM NPs	GNC	MMP-2; Acidic pH	FLimaging	PDT; DD	Xenograft bearing mice model	Xia et al., 2018
Au@BSA-NHA	AuNP	Hypoxia	CT		Xenograft bearing mice model	Shi et al., 2016
Au-PCM-AIDH	AuNC	Hypoxia		PTT; Free Radicals		Shen et al., 2017
DOX-HZN-DTDP @ Au NPs-LA-PEG2000-CAI	AuNP	Hypoxia Acidic pH		DD	Spheroid	Shabana et al., 2018

*AuNP, spherical gold nanoparticles; AuNR, gold nanorods; AuNC, gold nanocages; SERS imaging, Surface-enhanced Raman scattering imaging; PTT, photothermal therapy; FLimaging, Fluorescence imaging; MRI, magnetic resonance imaging; PAI, photoacoustic imaging, PDT, photodynamic therapy; MMP, matrix metalloproteinases; MMP-2, matrix metalloproteinases; NIRF tomografic imaging, near infrared fluorescence imaging; CT, computed tomography; DD, drug delivery; DOX, doxorubicin; PCM, phase-change material*.

### Tackling the Acidic pH in the TME

Several features of TME can be used to target nanomedicines to the tumor site for improved diagnosis and/or therapy. For example, the acidic microenvironment may be used to trigger responsive nanomedicines that release the cargo upon pH stimuli. In this respect, citraconic amides are moieties sensitive to pH, being hydrolyzed and converted in positively charged primary amines that are suitable to act as pH switches. Interaction with positive and negative charged nanoparticles cause nanoparticle aggregation that could be used for photoacoustic imaging, amplifying the signal and blocking exocytosis. Nanosystems using the aggregation of AuNPs induced by citraconic amides have been used as a diagnostic tool (Song et al., 2016; Li et al., 2019). Song et al. (2016) showed that an agent containing these amides coupled to AuNPs accumulates specifically in cancer cells, leading to an *in vivo* signal amplification that is the double of that non-functionalized NPs (Song et al., 2016). Additional functionalization may be added to the nanoplatform to also carry a suitable chemotherapeutic drug, resulting in a theranostic agent (Song et al., 2016). Li et al. (2019) created AuNPs functionalized with 4-(2-(5-(1,2-dithiolan-3-yl)pentanamido)ethylamino)-2methyl-4-oxobut-2-enoic acid (LSC) that contain the citraconic amide moiety and a cyclic RGDyK peptide conjugated with 16-mercaptohexadecanoic acid (c(RGDyK)MHDA) that allows the active tumor-targeting due to the connection to overexpressed integrin αvβ3 receptors (Li et al., 2019). Using a tumor-xenograft mouse model, the conjugated molecules, LSC and c(RGDyK), allowed for longer circulation times and enhanced accumulation at the tumoral tissue, which then provided for optimized photoacoustic signals at low concentration. Similar approaches using fluorescence imaging (FL imaging) activated in the TME due to the pH-sensitive structures have also been proposed to assist diagnostics and biodistribution assessment (Lai et al., 2017; Tang et al., 2019).

A similar rational has been used to enhance the efficacy of photodynamic therapy (PDT) and photothermal therapy (PTT). Liu et al. (2018b) used mesoporous silica coated gold nanorods (AuNR@MSN) with 2,3-dimethylmaleic anhydride (DMA)-modified chitosan oligosaccharide-block -poly (ethylene glycol) polymer (CS(DMA)-PEG) as a pH sensitive polymer. In presence of acidic pH, the amide bonds between CS and DMA are broken and the RLA ([RLARLAR]2) peptide is exposed to facilitate cellular internalization and mitochondrial accumulation. In addition, AuNR@MSM is also loaded with indocyanine green (ICG), a photosensitizer. Since the pH trigger allows for tumor targeting and selective internalization, upon laser irradiation both therapeutic approaches are active: (i) PDT triggers the production of ROS that will hamper cell viability; and (ii) PTT leads to heat generation that kills cancer cells in a selective way. The combination of these two approaches allows an enhanced therapeutic effect and lower side effects due to the guided TME accumulation (Liu et al., 2018b). Other have developed a system relying on self-assembly AuNPs through dithiol-polyethylene glycol (HS-PEG-SH) molecules that allow NP cross-linking. AuNPs are functionalized also with Doxorubicin (DOX), bonded through the hydrazone-thiol group, and an epidermal growth factor (EGF) peptide to act as an active targeting moiety to cancer cells overexpressing the EGF receptor. Once in the TME, the acidic and redox environment disassembles the complexes, and hydrolysis of the hydrazone bonds release the DOX. Using an *in vivo* mouse model the self-assembled AuNPs presented an increased accumulation at the TME when compared to solo AuNPs (Feng et al., 2017).

pH induced aggregation of metal nanoparticles may also be used to enhance the efficacy of photothermal therapy (Li et al., 2014; Dai et al., 2015). Similar to the concept described above, Jung et al. (2013) used citraconic amide moieties to trigger NP aggregation when within the acidic TME that leads to the creation of hot spots for surface-enhanced Raman scattering (SERS) imaging and shifts the absorption to the near infrared (NIR) for concomitant PTT (Jung et al., 2013). Another strategy that is based on TME induced NP aggregation was assayed for simultaneous photoacoustic imaging (PAI) and PTT (Yu et al., 2017). In this work, two types of AuNPs coated with complementary single-strand DNA containing at the pyridine-2-imine end an α-cyclodextrin (α-CD) were used. The acidic TME leads to the protonation of pyridine-2-imine triggering separation of the α-CD and allows aggregation of AuNPs due to complementary base pairing. Aggregation also favors the retention of AuNP at the TME, which can then be used for selective intratumoral PAI and PTT (Yu et al., 2017). Another approach based on the pH within the TME combined PDT and a set of imaging techniques: fluorescence, PAI, and magnetic resonance imaging (MRI) using core-shell gold nanocages@manganese dioxide (AuNC@MnO_2_) (Liang et al., 2018). The acidic TME triggers degradation of the MnO_2_ shell, releasing O_2_ and Mn^2+^, which causes an enrichment of the environment (including the hypoxic parts) with O_2_, thus potentiating the PDT. Upon irradiation, the presence of O_2_, Mn^2+^ and the AuNC itself allows for concomitant use of PDT, PAI, MRI, and FL imaging, in what is a multifunctional nanotheranostics. Besides the effective production of ROS due to the PDT that causes cell death, the events unleashed via this approach cause immunogenic cell death through the damage-associated molecular patterns (DAMPs) liberation and, consequently, dendritic cells maturation, which leads to effector cells activation (e.g., CD8 T cells, CD4 T cells, and NK cells). Once again, the response to the pH stimulus permits selective TME targeting, improving efficacy while decreasing undesired deleterious effects to neighboring healthy tissues.

Gold nanorods (AuNR) coated with mesoporous silica capped with chitosan have been used as PAI contrast agent for Multispectral Optoacoustic Tomography (MSOT) after NIR excitation (Zeiderman et al., 2016). For active targeting, these nanoconjugates were grafted with a pH sensitive variant 7 pHLIP peptide that allows specific accumulation at the tumor site. These AuNRs may also convey a cargo of chemotherapy (gemcitabine, a pyrimidine analog), where the TME acidic pH protonates the amino groups of chitosan, causing chitosan swelling (proton sponge) with consequent gemcitabine dissociation and release from the mesoporous silica (Zeiderman et al., 2016).

### The Case for the Matrix Metalloproteinases (MMP)

The increased expression of MMPs have an important role in tumor progression and the substrate of these proteinases can be used to selectively target nanomedicines to the TME. For example, these substrates conjugated to dyes coupled to AuNPs have been used as probes for imaging of cancer sites toward diagnostic applications (Wang et al., 2012). Since MMP-2 and MMP-7 are overexpressed in multiple tumors, the authors designed AuNPs functionalized with a peptide spacer that contains an MMP-2 substrate (Gly-Pro-Leu-Gly-Val-Arg-Gly) and an MMP-7 substrate (Val-Pro-Leu-Ser-Leu-Thr-Met-Gly). The N-terminus of the peptide has attached to a lanthanide complex, BCTOT-EuIII (BCTOT = 1,10-bis(5′-chlorosulfo-thiophene-2′-yl)-4,4,5,5,6,6,7,7-octafluorodecane1,3,8,10-tetraone) and the C-terminus has attached to a 7-amino-4-methylcoumarin (AMC), which may be hydrolyzed by one or both MMPs within the TME, and fluorescence emission of dyes occurs. This results in differences in fluorescence emission between cancer and normal cells that may be used for imaging (Wang et al., 2012). Lee et al. (2008) functionalized AuNPs with Cy5.5 dye attached to a peptide (Gly-Pro-Leu-Gly-Val-Arg-Gly-Cys) that contains the substrate for MMP (Pro-LeuGly-Val-Arg). Once in the TME, the substrate is recognized and cleaved by the proteases, causing dequenching of the dye and the near infrared fluorescence (NIRF) signal may be assessed via NIRF tomographic imaging (Lee et al., 2008). Sun et al. (2011) also used Cy5.5 dye linked to the same substrate for MMP but in a different peptide to develop a computed tomography (CT) contrast probe that may simultaneously be assessed by NIRF. The AuNPs were coupled with glycol chitosan polymers, which have shown excellent stability and tumor targeting ability by EPR, and an organic dark quencher. Upon MNP proteolysis, the dye is released and NIR fluorescence emission may be detected. As such, it is possible to obtain CT and NIRF images to provide anatomical and MMP-dependent biological data of the TME (Sun et al., 2011).

Gelatin is another substrate for MMP, particularly for MPP-2. Ruan et al. created a system for drug delivery relying on a gelatin nanoparticle decorated with small AuNPs (Ruan et al., 2015b). The AuNP surface is loaded with DOX linked by hydrazone bonds that are hydrolyzed when in the acidic TME medium. When the G-AuNPs-DOX-PEG nanoconjugate reaches the TME, the gelatin nanoparticles are degraded by the MPP-2, and the AuNPs-DOX-PEG released into the microenvironment. Once free, these smaller nanocarriers may reach deeper into the tumor and, due to the acidic microenvironment, DOX is released leading to a more efficacious anti-tumor effect. In another study, the same system was improved by addition of two components: a tandem peptide, RRGD, and Cy5.5 dye attached by hydrazone bonds to the AuNPs' surface. The RRGD peptide allows the active targeting to overexpressed integrin αvβ3 receptor and enhances the penetration capability of the system. The Cy5.5 is release together with DOX by hydrolyze of the hydrazone bonds in the acidic TME, permitting the visualization via fluorescence imaging. The released small AuNPs are capable of deeper tissue penetration, leading to accumulation at the target and colocalization of Cy5.5 and DOX, in what can be considered a two-stage multifunctional nanotheranostics system (Ruan et al., 2015a).

Relying on the fluorescence emission of DOX, Chen and co-workers developed a nanotheranostics system using AuNPs with DOX attached to the surface via a protease substrate (Ac-Cys-Pro-Leu-Gly-Leu-Ala-Gly-Gly-DOX) (Chen et al., 2013). Within the TME, MMP-2 cleaves the ligation and DOX is released, exerting its cytotoxic effect while proving for fluorescence imaging selectively within the TME. Another nanotheranostics system combining two therapeutic modalities, PDT and chemotherapy, coupled to fluorescence imaging has been developed using gold nanoclusters conjugated with an MMP-2 substrate (Cys-Pro-Leu-Gly-Val-Arg-Gly-Arg-Gly-Asp-Ser), and DOX bonded to cis-aconitic anhydride and a photosensitizer chlorin e6 (Ce6) (Xia et al., 2018). The MMP-2 substrate acts as active targeting to the TME due to the overexpression of metalloproteinases and the cis-aconityl linkage allows for controlled release of DOX on site due to the tumor acidic hydrolyzed of the bonds. The photosensitizer allows for PDT and imaging through fluorescence. The effect of this nanoconjugate in tumor-bearing mice show great promise, showing an enhanced anti-tumor effect when compared to free DOX and AuNPs without the MMP-2 substrate.

### Impact of ECM Components

The increased desmoplasia that frequently occur at the TME may result in poor distribution of AuNPs inside the tumor (Whilhelm et al., 2016). In an attempt of improving drug delivery into the tumor, Abdolahinia et al. analyzed the effect of AuNPs conjugated with collagenase (Col-AuNPs) combined with AuNPs conjugated with metformin (MET-AuNPs). They observed an increased number of apoptotic cells in breast cancer spheroids when simultaneously treated with Col-AuNPs and MET-AuNPs, suggesting increased AuNPs penetration in the spheroid (Abdolahinia et al., 2019). In another interesting study, Han et al. developed gold nanoparticles coated with PEGylated polyethylenimine and conjugated with all-trans retinoic acid (ATRA) and siRNA targeting heat shock protein 47 (HSP47) envisaging the activation of pancreatic stellate cells (PSC). With this pH responsive nanosystem, they were able to induce PSC quiescence (through ATRA) and inhibit ECM desmoplasia (through silencing of HSP47, a collagen-specific molecular chaperone) (Han et al., 2018). Interestingly, a study performed by Zhao et al. revealed that naked AuNPs were effective in decreasing the TME desmoplasia of colorectal cancer xerograft mice, by reducing the production of collagen I and diminishing the expression of profibrotic signals (Zhao et al., 2018).

Hyaluronic acid, a linear anionic polymer found in the connective, epithelial, and neural tissues has been widely used in the clinics for arthritis treatment, ophthalmic surgery, tissue engineering, and even drug delivery (Oh et al., 2010; Kim et al., 2019). Due to the presence of carboxylic acid, hydroxyl and N-acetyl groups, HA is easily combined with other chemicals. Moreover, the selective transfer of HA to the tumor location due to EPR effect, coupled to its connection with TME cellular receptors, including cluster determinant CD44, receptor for HA-mediated motility (RHAMM), and lymphatic vessel endothelial receptor-1 (LYVE-1), makes this molecule suitable for improving targeted nanomedicine (reviewed in Kim et al., 2019 and references therein). More recent reports describe the production of nanoconjugates for combined photothermal therapy (Liu R. et al., 2019; Yang et al., 2019). Liu et al. proposed to use cationic small sized red emission gold nanoclusters coated with BSA and conjugated with indocyanine green as imaging probe for theranostic and HA for increased retention at the tumor location (AuNC@CBSA-ICG@HA) (Liu R. et al., 2019). An *in vivo* study using mice breast cancer model revealed a suppression of 95% of tumor growth (Liu R. et al., 2019). Yang et al. proposed a nanotheranostics platform based on gold nanoclusters combined with graphene oxide and conjugated with HA, 5-fluorouracil. The enzymatic degradation of HA at the TME by the hyaluronidases allow the release of 5-fluorouracil, that, together with laser irradiation, enhance the anti-tumor efficacy (Yang et al., 2019).

### Making Use of Hypoxia for Selective Targeting to the TME

The hypoxic TME has an active role in tumorigenesis and has been shown to be a crucial factor in the lack of response to some therapies and cancer drug resistance. As such, it may be considered both as a critical element for selective action within the TME and as an important target for therapy and diagnostics. For example, Shi and co-workers presented a gold nanoprobe for CT imaging responsive to hypoxia through the conjugation of nitroimidazole moiety to AuNPs (Shi et al., 2016). Under hypoxia, the nitro group of nitroimidazole is reduced by nitroreductase, an enzyme present at elevated levels in the TME, and the resultant reactive amide group will bind to macromolecules within the TME leading to on site accumulation. The AuNPs are coated with bovine serum albumin (BSA) for increased colloidal stability, water solubility and biocompatibility. This technique enhances the contrast attained in CT imaging allowing for visualization of different intratumoral hypoxic levels which can be helpful to disease prognosis. Others have used AuNCs loaded with 2,2′-azobis[2-(2-imidazolin-2-yl) propane] dihydrochloride (AIPH) coated by a phase-change material, which allows the hypoxic cancer cells death through the oxygen independent production of free-radicals (Shen et al., 2017). After NIR irradiation, the photothermal effect of AuNC triggers the melting of the coating and, consequent AIPH release. Once free, AIPH decomposes due to thermal/irradiation stimulation and alkyl radicals are generated, that in turn cause oxidation of cell components oxidation and increased lipid hydroperoxides, which will induce apoptosis. This concept allows for the destruction of cells in hypoxic tumors and can be applied in combination with photothermal therapy. Shabana et al. took advantage of carbonic anhydrase IX (CA IX) overexpression that occurs in hypoxic TME to develop AuNPs functionalized with DOX and a CA inhibitor ligand, that allows active targeting to hypoxic tumors (Shabana et al., 2018). DOX is grafted to the NPs through a hydrazone group that is cleaved in acidic pH, releasing the drug. This way, it is possible to selectively target the TME to tackle the hypoxia-induced chemoresistance with improved tumor penetration of the drug.

### Synergistic Approach With the TME Immune System

Immune cells in the TME play an important role in tumor surveillance and development. The immune system behaves differently within the TME and its differential modulation also modifies the TME itself and can thus be used to improve therapy. Some strategies have been described that use AuNPs to modulate the immune system in the TME. For instance, AuNPs coated with mouse serum albumin (MSA) induce the production of ROS and reactive nitrogen species (RNS) that activate pro-inflammatory pathways in TAMs (Pal et al., 2016). As a consequence, the production of TNF-α and IL-10 is decreased and the production of the pro-inflammatory cytokine IL-12 increased. These changes triggered by AuNPs lead to a polarization of TAMs from an M2 phenotype to an M1 phenotype (pro-inflammatory). These changes in TAMs were also observed for a gold-manganese oxide nanocomposite stabilized with MSA, where the manganese oxide permits the enhancement of magnetic resonance of the agent and, as a result, allows its use as a contrast agent in magnetic resonance imaging (MRI). In another approach, AuNPs functionalized with tumor necrosis factor-related apoptosis-inducing ligand (TRAIL) that belongs to tumor necrosis factor (TNF) superfamily have also been used to actively target M2 macrophages within the TME (Huang and Hsu, 2017). The AuNPs bind to M2 macrophages due to the binding of TRAIL to the cell surface death receptor 4 (DR4) and death receptor 5 (DR5), which activates a caspase-dependent extrinsic apoptosis pathway and, consequently, cancer cell death in a specific way. AuNPs have also been used as a carrier for tumor-associated self-antigens that elicit the maturation of dendritic cells and T cells proliferation (Ahn et al., 2014; Fogli et al., 2017). Also, galactofuranose-coated AuNPs demonstrated the capability to stimulate the maturation of dendritic cells and, thus, promote a pro-inflammatory response (Chiodo et al., 2014). The modulation of TAMs, dendritic cells and T cells indicate to be suitable strategies to modulate the immune response within the TME against cancer cells so as to hamper tumor progression.

### Addressing Angiogenesis

The growth of new blood vessels from preexisting vessels is essential for tumor expansion and to modulate TME, increasing the levels of oxygen, nutrients, and decreasing toxic metabolites (De Palma et al., 2017). In this respect, AuNPs have been shown to be capable not only to disrupt signal transduction from tumor mesenchymal cells to epithelial cells, but also to inhibit angiogenic phenotypes *in vitro* (Zhang Y. et al., 2019). Among the various VEGF subtypes, isoform VEGF165 is a heparin binding protein and perhaps the most potent cytokine in angiogenic process (Mukherjee et al., 2005; Arvizo et al., 2011). By binding to tyrosine kinase receptor VEGFR-2, a signal cascade is initiated, ultimately leading to the proliferation and migration of endothelial cells and resulting in angiogenesis. As such, therapeutic antibodies targeting VEGF165 have been developed and are currently applied in the clinics to inhibit the VEGF165 cascade activation (Ferrara and Kerbel, 2005). Also, non-functionalized AuNPs are capable to inhibit of pro-angiogenic heparin-binding growth factors (HB-GFs), such as VEGF165, basic fibroblast growth factors (bFGFs), and placental growth factor (PlGF) (Ferrara and Kerbel, 2005). The surface of AuNPs inhibits HB-GFs by inducing changes in conformation but, remarkably, AuNPs do not affect the activity of non-HB-GFs, such as VEGF121 and EGF. These observations have been corroborated *in vivo*, where VEGF/vascular permeability factor (VPF) stimulated permeability was inhibited by AuNPs (Mukherjee et al., 2005; Arvizo et al., 2011). AuNPs may also influence angiogenesis by diminishing the tube formation and the migration of endothelial cells through blockage of the VEGF-VEGFR2 signaling in TME cells (Zhang Y. et al., 2019). What is more, AuNPs have been able to promote tumor vasculature normalization while increasing blood perfusion and reducing hypoxia (Li et al., 2017).

Not only “naked” AuNPs but also AuNP-conjugates have been used as modulators of angiogenesis. Recombinant human endostatin is an anti-angiogenic agent used for tumor treatment that has been coupled to AuNPs to reduce cell migration and tube formation *in vitro* (HUVECs), induced by anterior gradient 2 (AGR2) (Pan et al., 2017). AGR2 is one of the latent tumor angiogenesis factors, which has been mostly related to tumor cell proliferation, transformation, migration and drug resistance. *In vivo* metastatic colorectal cancer xenografts have shown that recombinant human endostatin–AuNP are capable to increase pericyte expression while inhibiting VEGFR2 and anterior gradient 2. As such, these nanoconjugates might be used to normalize tumor vessels using AGR2 as an anti-angiogenic tumor target. Additional work by the Kanaras group has demonstrated the enhanced effect of peptides, designed to selectively interact with VEGFR1, for inhibition of angiogenesis when grafted onto AuNPs (Bartczak et al., 2013, 2015; Millar and Kanaras, 2013). This gold-nanoconjugates can influence the extent and morphology of vascular structures, without causing toxicity (Bartczak et al., 2013, 2015; Millar and Kanaras, 2013). By exposing a chicken chorioallantoic membrane to the same AuNP-peptide, the same effect was observed *in vivo* (Roma-Rodrigues et al., 2016), but could be tremendously enhanced by irradiating the nanoformulation with a green laser, which simultaneously allowed to cauterize emerging blood vessels with extreme spatial precision, and prevented neo-vascularization (Pedrosa et al., 2017). These studies highlighted the role of the VEGFR1 dependent pathways in the process, which were downregulated by the nanoformulation with and without irradiation (Pedrosa et al., 2017). These studies show that AuNPs can be used to alter the expression of anti-angiogenic factors, under biological conditions, and may be a valuable tool to tackle the angiogenesis within the TME.

### The Role for TME Derived Exosomes

The role of exosomes in TME maturation combined with their stability in body fluids, have made these nanovesicles as promising agents in cancer management. There have been several studies reporting on the use of AuNPs for therapy and diagnostics (nanotheranostics) (see Roma-Rodrigues et al., 2017b for extended discussion). The developmental and metabolic state of each cell is reflected on exosome production, trafficking and the exosomes' own content. What is more, after entering the circulatory system, TME derived exosomes have been proposed as valuable cancer biomarkers, particularly when referring the use of liquid biopsies. Nevertheless, the direct diagnostics using exosomes, is hampered by the low concentration of circulating tumor cells derived exosomes (TCDEs) in the initial stages of tumor progression. As such, Huang and co-workers designed a dual-signal amplification platform for detection of exosomes derived from leukemia cells (Huang et al., 2018). The detection system consisted in three steps: (i) the high abundance of the tetraspanin CD63 at the exosome membrane was used to capture the vesicles using anti-CD63 antibodies conjugated to magnetic beads; (ii) the highly abundant nucleolin at the surface of exosomes was used as target to bind the vesicles to a nucleolin-recognition aptamer (AS1411), allowing the initiation of a rolling circle amplification (RCA) reaction; and (iii) the resulting amplified sequences hybridize with AuNPs conjugated with a specific oligonucleotide and a quenched fluorescent dye (FAM), which, via the action of an endonuclease, will result in FAM release and concomitant emission of fluorescence. With this platform, it was possible to detect exosomes in a spiked serum sample with high sensitivity. Others have used a SERS-based biosensors by modifying the surface of gold nanostars@4-mercaptobenzoic acid@nanoshell structures with bivalent cholesterol- labeled DNA anchor (Tian et al., 2018). After capturing exosomes with magnetic beads containing CD-9 antibody, the SERS biosensors were fixed at the exosome surface through hydrophobic interactions between the cholesterol present in the beads and the lipidic membrane of the vesicles. When assembled to the exosomes, a SERS signal could be attained for improved detection. Also focused on SERS, a screening platform using capturing substrates, consisting in gold shell magnetic nanobeads conjugated with aptamers for recognition of CD63 at the surface of exosomes, and in SERS probes with three distinct Raman reporters was proposed (Wang Z. et al., 2018). With this technology, it was possible to detect distinct types of exosomes in blood samples, rendering this methodology as promising for diagnosis of early stage cancer.

Also, one the most important characteristics of exosomes is their role in transport of virus, chemotherapeutic agents, DNA and proteins, making them promising vectors for cancer therapy (reviewed in Srivastava et al., 2016). Recent studies describe the transport of AuNPs by exosomes derived from different types of cells, including macrophages and breast cancer cells (Roma-Rodrigues et al., 2017a; Logozzi et al., 2019). Taking advantage of this feature, Sancho-Albero et al. loaded MSCs with hollow AuNPs and revealed a selective transfer of the exosome-cargo into cells of the same type than that of the cell of origin. It was then proposed to apply this preferential uptake conjugated with light-induced hyperthermia for anti-cancer therapy (Sancho-Albero et al., 2019).

### Taking Advantage of Folate Requirements of Tumor Cells for Active Targeting

As already mentioned in section Tumor Microenvironment, the high proliferation rate of tumor cells demands a higher nutritional supply, resulting in the increased expression of nutrient receptors, such as folic acid receptors (or folate) to respond to the high request of folate for DNA synthesis (Farran et al., 2019). The functionalization of AuNPs with folate has showed a great potential for increased AuNPs targeting in cancer (reviewed in Samadian et al., 2016; Beik et al., 2017). Several groups have proposed gold nanostructures functionalized with PEG conjugated with folate for photothermal therapy (Ghaznavi et al., 2018; Wang J. et al., 2018; Majidi et al., 2019). Ghaznavi et al. (2018) developed gold and iron oxide core-shell nanoparticles coated with PEG conjugated with folate (FA-PEG-Au@IONP), Majidi et al. synthesized silica and gold core-shell nanoparticles with folate (FA-SiO_2_@AuNPs) and Wang et al. proposed a gold nanostars-based nanocomposites conjugated with a fluorescent polypeptide for image-guided therapy. Zeinizade et al. analyzed the effect of folate conjugated AuNPs. In these four reports, increased apoptosis was observed only when tumor cells were simultaneously treated with nanoconjugates and laser irradiation (Ghaznavi et al., 2018; Wang J. et al., 2018; Zeinizade et al., 2018; Majidi et al., 2019). With the same objective, Wang et al. designed multi-layered single walled carbon nanotubes consisting in single walled carbon nanotubes coated with BSA and functionalized with PEG conjugated with folate and with doxorubicin (SWNT@BSA@Au-S-PEG-FA@DOX). The treatment of mice bearing tumors with nanoconjugates and near infrared laser (808 nm) resulted in complete tumor eradication with negligible damage of normal tissues (Wang D. et al., 2018). Zhao et al. developed gold nanochains with worm like structures as a light-triggered system for photodynamic therapy and multiplex detection. These gold nanochains were synthesized using hyaluronic acid-hydrocaffeic acid conjugates as templates and Raman reporters, photosensitizers and folate for active targeting. They observed that low concentrations of these nanoconjugates result in high selectivity and phototoxicity after laser irradiation (Zhao et al., 2019).

Other groups have proposed gold-based nanocomposites conjugated with folate for imaging purposes. Beik et al. analyzed with detail the effect of AuNPs conjugated with folate for CT with the advantage of using lower dosage and enhanced image contrast (Beik et al., 2018). Kumar et al. used green synthesis to prepare gum kondagogu capped gold nanoparticles coupled to folate and fluorescein isothiocyanate (FITC) and observed a high affinity of nanoconjugates toward folate receptor positive cells (Kumar et al., 2018). In another approach, Zhang et al. synthesized mesoporous carbon-gold hybrid nanozyme nanoprobes stabilized with BSA and folate and loaded with IR780 iodide (OMCAPs@rBSA-FA@IR780). *In vivo* analysis revealed efficient tumor targeting and retention (Zhang A. et al., 2019). Khademi et al. studied the *in vivo* targeting of AuNPs conjugated with folate through cysteamine (FA-Cys-AuNPs) for nasopharyngeal head and neck cancer, which were judged suitable for use as contrast agents in CT scan imaging (Khademi et al., 2019).

## Conclusions

In the past, cancer, and tumor development have been considered alone almost as if a strange body to the host organism. Nowadays, tumorigeneses and development must be considered as an element within a larger context that modulates itself in a continuous process of cell progression and maturation. The tumor micro-environment constitutes that differentiating niche where clonal expansion is possible through a multitude of molecular inputs that are at the basis of cancer heterogeneity. Notwithstanding the exponential development of therapeutic approaches to tackle these niches, there has been a growing need to combine several modes of action and therapy modalities simultaneously to tackle the TME. Nanomedicine has been providing for a plethora of multi-functional platforms capable of conveying different cargos to the site of interest in a selective way, while simultaneously providing for diagnostics, imaging and/or biodistribution capability, toward earlier detection, and destruction of cancer cells. These nanotheranostics systems have relied heavily on gold nanoparticles due to their chemical versatility, biocompatibility and unique spectral properties. This way, making use of the specific conditions within the TME, it has been possible to develop bio responsive platforms to selectively address this particular cellular context. AuNPs have been engineered so as to tackle and/or profit from particular aspects of the TME toward a more selective targeting of the tumor site, or a focal delivery of the therapeutic cargo, and/or to accumulate the nanoconjugates at a precise location that potentiates the use of photoinduced therapies based on the spectral properties of AuNPs.

Still, there are several issues that need to be addressed. Despite the growing number of *in vitro* modeling systems, these are still not equivalent to the real TME. Usually these *in vitro* models, be it the 3D co-cultures and/or on-chip platforms, do not address more than three to four types of cells embedded in an unchanging support media/scaffold. As such, one only gets a couple of *photograms* from the evolving context of the TME *film*. These photograms provide valuable information to address a particular factor and/or pathway in a hierarchized hypothesis rather than the *whole* TME. Nevertheless, the possibility to link and associate several of these models into more complex and interconnected chip systems may provide for systems that more closely resemble what is happening in the TME *in vivo*, and that researchers only now start to get a deeper understanding of events. These models, despite their actual limitations, have been providing for valuable tools to screen and develop new approaches to study and tackle the TME. Here, innovative nanomedicines have taken the lead in making use of the conditions and characteristics of the TME to deliver therapeutics with increasing precision, while providing for signal outputs that allow to follow their effect in real time—real nanotheranostics platforms.

These nanotheranostics strategies may be combined in approaches that simultaneously profit from distinct features of the TME for potentiating the active targeting, controlled release, and therapy modality coupled to imaging/diagnostics. As such, the combination of these features may provide for valuable tools to tackle the TME, addressing the tumor heterogeneity, decreasing tumor resistance, and bringing more efficacious strategies to the clinics.

## Author Contributions

PB: writing—concept. CR-R, LR, PP, IP, AF, and PB: original draft preparation and discussion. CR-R: artwork. AF and PB: review and editing.

### Conflict of Interest Statement

The authors declare that the research was conducted in the absence of any commercial or financial relationships that could be construed as a potential conflict of interest.

## Abbreviations

AGF: anterior gradient 2
AIPH: 2,2′-azobis[2-(2-imidazolin-2-yl) propane] dihydrochloride
AMC: 7-amino-4-methylcoumarin
AuNC: gold nanocage
AuNP: gold nanoparticle
AuNR: gold nanorod
AuNR@MSN: mesoporous silica coated nanorod
bFGF: basic fibroblast growth factors
BSA: bovine serum albumin
CAFs: cancer associated fibroblasts
CD: cyclodextrin
CS: chitosan oligosaccharide
CSC: cancer stem cell
CT: computed tomography
DAMPs: damage-associated molecular patterns
DMA: 2,3-dimethylmaleic anhydride
DOX: doxorubicin
ECM: extracellular matrix
EGF: epithelial growth factor
EMT: epithelial-to-mesenchymal transition
EPR: enhanced permeability retention
FL: fluorescence
GM-CSF: granulocyte-macrophage colony stimulating factor
HA: hyaluronic acid
HB-GFs: heparin-binding growth factors
HIF: hypoxic-induced factor
HM: humanized mice
HTS: high throughput screening
ICG: indocyanine green
IL: interleukin
LECs: lymphatic endothelial cells
LSC: 4-(2-(5-(1,2-dithiolan-3-yl)pentanamido)ethylamino)-2methyl-4-oxobut-2-enoic acid
MDR: multidrug resistance
MDSCs: myeloid-derived suppressive cells
MET: mesenchymal-to-epithelial transition
MMPs: matrix metalloproteinases
MRI: magnetic resonance imaging
MSA: mouse serum albumin
MSOT: multispectral optoacoustic tomography
NIR: near infrared
NIRF: near infrared fluorescence
NK: natural killer
OoC: organ-on-a-chip
PAI: photoacoustic imaging
PCM: phase-change material
PDC: patient derived xenografts
PDT: photodynamic therapy
PEG: poly-ethylene glycol
PlGF: placental growth factor
PTT: photothermal therapy
RCA: rolling circle amplification
RNS: reactive nitrogen species
ROS: reactive oxygen species
SERS: surface-enhanced Raman scattering
TAMs: tumor associated macrophages
TCDEs: tumor cells derived exosomes
TGF-β: transforming growth factor beta
TME: tumor microenvironment
TNF: tumor necrosis factor
ToC: tumor-on-a-chip
TRAIL: tumor necrosis factor-related apoptosis-inducing ligand
VEGF: vascular endothelial growth factor
VEGFR: vascular endothelial growth factor receptor
VPF: vascular permeability factor.
